# Roland Littlewood

**DOI:** 10.1192/bjb.2026.10212

**Published:** 2026-08

**Authors:** Maurice Lipsedge

Professor of Anthropology and Psychiatry, University College London, UK

**Figure:**
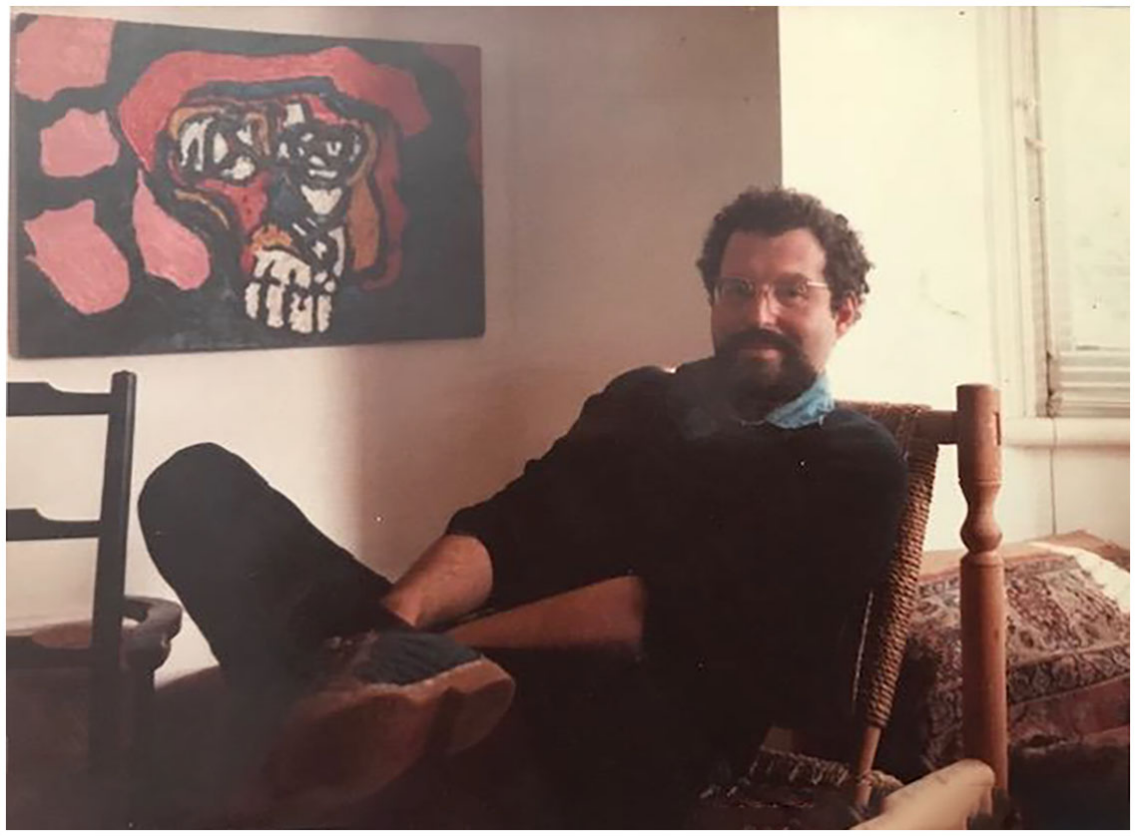

Roland Littlewood, psychiatrist and anthropologist, who profoundly influenced our understanding of culture and mental disorders, died in November 2025, age 78.

Roland was born in 1947 into an academic family in Leicester, where his father, Robert, was a lecturer in Spanish, and his Swiss mother, Gertrude Lehner, taught French and German. The family moved to the medieval village of Hallaton, renowned for its annual bottle-kicking tournament. Reckless as ever, Roland would always plunge into the mêlée.

Roland began his medical career at St Bartholomew’s Hospital (Bart’s). Founded in 1123, it was a conservative institution until the 1960s, when anti-establishment medical students and staff emerged. These included Graham Chapman of Monty Python, Martin Birnstingl, the vascular field surgeon who worked with the Medical Aid Committee for Vietnam treating the victims of napalm bombing, and Joseph Rotblat, a professor of physics who was awarded the 1995 Nobel Peace Prize for his dedication to the non-military uses of atomic energy.

My student Roland was another radical, a Marxist combatant in the 1968 Battle of Grosvenor Square, where he confiscated a policeman’s helmet. He was not a dilettante but a polymath, a scholar of both Sanskrit and semiotics, of Robert Musil and phenomenology. He had an honours degree in biochemistry and would regularly play truant from Bart’s to attend art classes at the Sir John Cass School of Art in Whitechapel. It was there that Roland developed his unique and audacious expressionistic style.

Although he failed the surgery exam in his final London MB BS, he pulled his socks up and completed his medical and surgical house jobs at Bart’s. While living in a tiny studio above a launderette, Roland became my senior house officer at F Block, the psychiatric unit at the near derelict Hackney Hospital. There was no privacy and no curtains around the beds. F Block was criticised for abysmal standards by the Hospital Advisory Service in the 1970s.

Roland thrived in this challenging environment, devising his own form of art therapy for psychotic patients. He would paint alongside them, discussing *their own* meanings of the paintings. In this, Roland followed a fellow Swiss, Eugen Bleuler, who established a rapport by interpreting in the local dialect what were previously dismissed as ‘senseless utterings’. (Years later, Roland served on the Academic Board of Goldsmiths College Art Therapy MSc.)

Most of our patients lived in London N16 – Stoke Newington – or N4 – Stamford Hill. They came from two distinct cultural minorities – African-Caribbean and ultra-orthodox Jewish. They inspired much of Roland’s early research, with papers on the over-diagnosis of schizophrenia in West Indians, and on extreme heterodox behaviour in ‘The antinomian Hasid’ (1983). Drawing on our clinical experience we wrote *Aliens and Alienists: Ethnic Minorities and Psychiatry*.^1^ Roland wrote most of this book, which went into three editions between 1982 and 1997. His scholarly understanding of the Hasidic community also inspired Simon Dein’s *Religion and Healing among the Lubavitch* (2004).

With Roland’s gift for cross-cultural dialogue, it was natural for him to train in social anthropology at Oxford and to carry out years of ethnographic fieldwork within a new religious community. Published in 1993, *Pathology and Identity* ^2^ described the millennial cult established by Prophetess Mother Earth in an abandoned village within ‘Hell Valley’, a remote area of north-east Trinidad. The founder’s hallucinations during an organic psychotic episode became systematised into a new religion that drew on West African themes to establish a radical and antinomian horticultural community. The Prophetess asserted female creativity and claimed that people of African origin are the custodians of Nature. She challenged the ecological threats from European science.

Roland’s monograph on ‘Hell Valley’ is sprinkled with irreverent asides. Describing the Prophetess Mother Earth’s ‘frank and radical identification with the Devil’, Roland concluded: ‘There is, quite simply, enormous pleasure in the rather delicious pursuit of “evil”.’^3^ Despite his snake phobia, Roland joined the Earth People in sleeping on the ground. As a participant in everyday life in the chiliastic village where there was ‘relatively little awe attached to Mother Earth as incarnate Nature’, Roland reflected: ‘On many occasions I wondered if the group should be seen less as a “religious” community than as some high-spirited summer camp.’^4^

His many books include *The Butterfly and the Serpent: Essays in Psychiatry, Race and Religion*.^5^ He also wrote about pathomimesis, Nafsiyat, Druze reincarnation, domestic sieges, the Albanian third sex, DSM-IV, the British National Party, zombification, sorcery as spandrel and multiple consciousness. The essence of Roland’s work as both anthropologist and psychiatrist was to recognise the significance of both the ‘personalistic’ and the ‘naturalistic’, by which he meant the humanities and the natural sciences. He taught that both are valid and necessary, and neither can be reduced to the other.

After only two years working as a consultant psychiatrist in a deprived part of inner-city Birmingham, Roland was awarded the Wellcome Medal for Anthropology as Applied to Medicine, in 1993. Just one year later he was appointed as the first Professor of Anthropology and Psychiatry at University College London. In the same year he was elected as President of the Royal Anthropological Institute. In 1999, Roland achieved FRCPsych and gave The Wilde Lectures in Natural Religion at Oxford. In 2002, he was awarded a DLitt for Social Anthropology by London University, and in 2005 a DSc in Medicine by Oxford University.

Roland married Jenny, a Bart’s nurse who went on to publish *Anthropology and Nursing*.^6^ His brother, Antony, is an authority on Byzantine literature. Roland’s unmatched sense of the absurd was expressed in both his dress and his writing. For a period, he was Aristide Bruant in a bright red scarf and dark overcoat. More recently he sported an Afghan Pashtoon bonnet.

Roland endured metastatic cancer of the pancreas with characteristic fortitude, and he was busy writing his latest book, *Between Anthropology and Psychiatry*, with Simon Dein, almost until the end. Jenny and their daughter, Letice, were with him on Saturday 22 November: Letice had just framed one of Roland’s extraordinary paintings.
